# L-Histidine Improves Boar Sperm Quality by Alleviating Oxidative Stress During Preservation at 17 °C

**DOI:** 10.3390/antiox15091086

**Published:** 2026-08-28

**Authors:** Qingzhe Meng, Yongjin Liu, Xiaohong Duan, Xifei Zhang, Guijiang Wang, Lingjiang Min, Eslam M. Bastawy, Fei Luo, Zhendong Zhu

**Affiliations:** 1 College of Animal Science and Technology, Qingdao Agricultural University, Qingdao 266109, China; 20242109037@stu.qau.edu.cn (Q.M.); 20242109056@stu.qau.edu.cn (Y.L.); mljlab405@qau.edu.cn (L.M.); 2 Hebei Livestock Breeding Station, Shijiazhuang 050060, China; duanxiaohong0810@163.com (X.D.); 18731751818@163.com (X.Z.); 13933077085@126.com (G.W.); 3 Faculty of Health, School of Medicine, Institute for Mental and Physical Health and Clinical Translation, Deakin University, Geelong 3216, Australia; eslam_bastawy@sci.asu.edu.eg

**Keywords:** L-histidine, boar sperm, antioxidant activity, sperm quality

## Abstract

Oxidative damage is closely associated with the gradual decline in boar sperm quality during liquid storage at 17 °C. To determine whether L-histidine (L-His) could limit this deterioration, semen was diluted with an extender containing 0, 1, 10, 100, or 1000 μM L-His. Motility and acrosomal integrity were measured throughout storage. On day 7, mitochondrial membrane potential (MMP) and ATP content were determined together with intracellular probe oxidation, membrane lipid oxidation, and Annexin V-FITC/PI staining patterns. The stored sperm were subsequently exposed to capacitating conditions and tested for their ability to bind to oviductal explants. The most favorable responses were observed with 100 μM L-His. Compared with untreated semen, this group retained higher motility and acrosomal integrity and showed higher MMP and ATP content (*p* < 0.05). It also exhibited less intracellular probe oxidation and membrane lipid oxidation, together with lower proportions of Annexin V-positive and membrane-compromised sperm. Following capacitation induction, sperm stored with 100 μM L-His showed increased tyrosine phosphorylation and higher proportions of capacitated sperm. Their binding index to oviductal explants was also higher than that of the control group. These findings indicate that supplementation with 100 μM L-His can improve the preservation quality of boar sperm during extended liquid storage at 17 °C and maintain functional characteristics of stored sperm, providing new insights into the potential use of L-His as a semen extender supplement for prolonged storage.

## 1. Introduction

Liquid-preserved semen is widely used for artificial insemination in commercial pig production [1,2]. Its application efficiency is largely determined by the ability to preserve sperm functionality during semen processing and storage [3]. During prolonged storage, boar sperm gradually deteriorate, and oxidative imbalance is considered an important contributor to this process. The abundance of polyunsaturated fatty acids in the plasma membrane leaves boar sperm susceptible to lipid peroxidation, particularly because their antioxidant defenses are limited [4,5]. When ROS accumulate during liquid storage, this imbalance can be accompanied by membrane injury, loss of mitochondrial membrane potential, altered cellular energy status, and apoptotic changes [6]. Accordingly, various antioxidants have been incorporated into semen extenders to alleviate oxidative damage during storage [7,8].

L-Histidine (L-His) is an essential amino acid containing an imidazole side chain [9]. The imidazole group of L-His contributes to its biological activity through pH-dependent chemical properties [10,11]. L-His also serves as a precursor for carnosine synthesis together with β-alanine [12]. The imidazole ring enables histidine and related compounds to quench singlet oxygen and react with other oxidants. Even so, histidine is a weaker direct radical scavenger at physiological pH than cysteine, tyrosine, or tryptophan [13,14]. The same imidazole group can also form covalent adducts with electrophilic products of lipid peroxidation, including 4-hydroxynonenal (4-HNE). This property has recently been considered in the optimization of preservation media for human sperm [15]. Carnosine, a histidine-containing dipeptide synthesized from β-alanine and histidine, has been reported to attenuate lipid oxidation and carbonyl-associated damage, suggesting a potential protective role of histidine-derived compounds under oxidative stress conditions [16,17].

Evidence concerning the effects of L-His on male reproductive function and sperm remains limited. In a lead-exposed rat model, histidine supplementation alleviated testicular oxidative stress and mitochondrial dysfunction and was associated with improvements in spermatogenesis and sperm parameters [18]. In addition, histidine was included in an optimized medium that supported the prolonged maintenance of human sperm motility, possibly because of its ability to limit oxidative and lipid aldehyde-associated damage [15]. Nevertheless, whether L-His can protect boar sperm during liquid storage has not yet been established.

Sperm function during liquid storage depends on the maintenance of membrane integrity and mitochondrial status in addition to motility [19]. Oxidative damage to these cellular components may compromise sperm survival and subsequent fertilization-related functions [20]. Therefore, assessing the effects of L-His requires consideration of oxidative status, mitochondrial characteristics, and post-storage functional properties in addition to conventional sperm quality parameters.

To examine the effect of L-His during liquid storage, boar semen was stored at 17 °C with different concentrations of L-His. Sperm quality, oxidative status, mitochondrial membrane potential, ATP content, and sperm apoptosis were assessed during or after storage. After 7 days, we also evaluated thermo-resistance, capacitation-related changes, and sperm binding to oviductal explants. The novelty of the present study lies in evaluating the potential use of L-His as a semen extender supplement for prolonged liquid storage and its ability to maintain the preservation quality and functional characteristics of boar sperm.

## 2. Materials and Methods

### 2.1. Ethical Aspects

All procedures were approved by the Animal Use Ethics Committee of Qingdao Agricultural University and conducted in accordance with its guidelines (protocol no. DKY20240814).

### 2.2. Chemicals

Unless otherwise specified, all chemicals and reagents were purchased from Sigma-Aldrich (Shanghai, China).

### 2.3. Extender Preparation

The Modena extender was prepared as described in our previous study [21] and contained 153 mM D-glucose, 26.7 mM trisodium citrate, 11.9 mM sodium bicarbonate, 15.1 mM citric acid, 6.3 mM disodium ethylenediaminetetraacetate (EDTA-2Na), 46.6 mM Tris, and 1000 IU/mL penicillin G sodium salt.

### 2.4. Semen Collection

Five healthy, sexually mature Duroc boars (20.3 ± 3.5 months of age) were used as semen donors. The boars were housed individually and fed 2.5 kg per day of a commercial diet formulated for breeding boars, divided into two meals, with water available ad libitum. The sperm-rich fraction of each ejaculate was collected weekly using the gloved-hand technique and filtered through two layers of sterile gauze. At each collection, the filtered sperm-rich fractions from the five boars were pooled to minimize individual variation. Three pooled semen samples obtained from three independent weekly collection sessions were used as biological replicates, with each pooled sample serving as one experimental unit.

### 2.5. Semen Processing and Storage

Each pooled semen sample was divided into five aliquots and diluted with Modena extender containing 0 (control), 1, 10, 100, or 1000 μM L-His (H0020, Lot No. 24231127003, Beijing Solarbio Science & Technology Co., Ltd., Beijing, China; molecular weight: 155.13 g/mol) to achieve a final sperm concentration of 3 × 10^7^ sperm/mL. The diluted semen samples were stored at 17 °C in a temperature-controlled refrigerator and evaluated on days 1, 3, 5, and 7 of storage.

### 2.6. Assessment of Semen Motility

On days 1, 3, 5, and 7 of liquid storage at 17 °C, a 1 mL aliquot of each treatment sample was collected for motility assessment. Sperm motility and kinematic parameters were evaluated using a computer-assisted sperm analysis system (CASA; CEROS II, Hamilton Thorne Inc., Beverly, MA, USA), as described previously [22]. The evaluated parameters included total motility (TM), progressive motility (PM), curvilinear velocity (VCL), straight-line velocity (VSL), average path velocity (VAP), straightness (STR), linearity (LIN), wobble (WOB), amplitude of lateral head displacement (ALH), and beat-cross frequency (BCF). For each treatment within each pooled semen collection, six randomly selected fields containing at least 200 sperm per field were analyzed. The field-level values were averaged to obtain one value for each treatment within each biological replicate. Three independent pooled semen collections were used as biological replicates for CASA.

### 2.7. Assessment of Viability and Acrosome Integrity

Sperm viability and acrosomal integrity after 1, 3, 5, and 7 days of storage were assessed by FITC-PNA/PI staining as described in our previous study [23]. Briefly, 0.6 μL of FITC-PNA (1 mg/mL) and 0.54 μL of PI (2.4 mM) were added to 1 mL of diluted semen, and the samples were incubated at 37 °C in the dark for 5 min. FITC-PNA binds specifically to glycan residues that become exposed upon acrosome damage. Therefore, sperm with compromised acrosomes exhibit green fluorescence due to FITC-PNA binding. PI is a membrane-impermeable dye that can only penetrate sperm with damaged membranes, resulting in red fluorescence in membrane-compromised or non-viable sperm. Samples were analyzed using a flow cytometer (BeamCyte, Changzhou, China); a total of 20,000 sperm events were analyzed. Sperm negative for FITC-PNA fluorescence were classified as having an intact acrosome, whereas sperm negative for PI were considered to possess an intact membrane. Sperm negative for both FITC-PNA and PI (FITC-PNA−/PI−) were classified as viable sperm with intact acrosomes. The percentage of viable sperm with intact acrosomes was calculated relative to the total sperm population and used for statistical analysis. This assay was performed using three independent pooled semen collections. Flow cytometric events were used to estimate the percentage of each sperm population and were not treated as independent biological replicates. Flow cytometry data were processed and analyzed using FlowJo software (v10.8.1; FlowJo LLC, Ashland, OR, USA).

### 2.8. Assessment of Mitochondrial Membrane Potential (MMP)

MMP in sperm stored for 7 days was assessed using the fluorochrome JC-1 dye (65-0851-38, Thermo Fisher Scientific, Boston, MA, USA), as described in our previous study [22]. A positive control was prepared by adding 0.2 μL of a 10 mM CCCP solution to 200 μL of control semen, followed by incubation at 37 °C for 20 min. Then, 200 μL of each treatment sample was mixed with 400 μL of JC-1 working solution. The positive control was processed similarly. The JC-1 working solution was prepared by combining 50 μL of 200× JC-1 reagent with 8 mL of water and 2 mL of 5× JC-1 staining buffer. All mixtures were subsequently incubated at 37 °C for 20 min before flow cytometer analysis. Sperm with high MMP were indicated by the formation of JC-1 aggregates, whereas sperm with low MMP were indicated by JC-1 monomers. Flow cytometry data were processed and analyzed using FlowJo software (v10.8.1). The experiment was performed using three independent pooled semen collections as biological replicates. Flow cytometric events were used to estimate the proportion of sperm with high or low MMP and were not treated as independent biological replicates.

### 2.9. Assessment of Sperm ATP Levels

Intracellular ATP levels in sperm stored for 7 days were quantified using a commercial ATP Assay Kit (ADS-W-A001-96, AIDISHENG, Yancheng, China) according to the manufacturer’s instructions and the method described in our previous study [23]. Briefly, 1 mL of sperm suspension from each treatment group was centrifuged, and the resulting pellet was resuspended and homogenized to lyse the sperm cells. An aliquot of the homogenate was collected for the determination of total protein concentration. The remaining suspension was boiled for 10 min to denature proteins, vortexed for 1 min, and subsequently centrifuged to remove debris. ATP levels were measured following the manufacturer’s instructions. The supernatant sample and assay reagents were added sequentially to a 96-well microplate, and absorbance was recorded using a microplate reader (Infinite M Nano, Tecan, Männedorf, Switzerland) upon completion of the reaction. ATP concentrations were normalized to total protein content for each sample. ATP measurements were performed using three independent pooled semen collections as biological replicates.

Protein concentration was determined using a BCA Protein Assay Kit (GK10009, GLPBIO, Montclair, CA, USA) according to the manufacturer’s instructions. Briefly, BCA working reagent was prepared by mixing Reagent A and Reagent B at a ratio of 50:1. Then, 25 μL of each standard or protein sample was added to a 96-well microplate, followed by 200 μL working reagent. After incubation at 37 °C for 30 min, absorbance was measured at 562 nm using a microplate reader. Protein concentrations were calculated according to the standard curve.

### 2.10. Assessment of Membrane Lipid Oxidation

Membrane lipid oxidation in sperm stored for 7 days was assessed using BODIPY 581/591 C11 (Molecular Probes, Eugene, OR, USA), following the method described in our previous study [24]. This membrane-incorporating probe emits red fluorescence in its reduced form and shifts toward green fluorescence following oxidation. Briefly, 200 µL aliquots of sperm suspension were washed twice by centrifugation at 200× *g* for 5 min. The pellets were resuspended and incubated with BODIPY 581/591 C11 at a final concentration of 2 µM for 30 min at 37 °C in the dark. After staining, the samples were centrifuged at 800× *g* for 5 min to remove the unbound probe and immediately analyzed by flow cytometry. A total of 20,000 sperm-specific events were recorded per sample, and the data were processed using FlowJo software (v10.8.1). Three independent pooled semen collections were used as biological replicates, and individual flow cytometric events were not treated as independent replicates.

### 2.11. Assessment of Intracellular Oxidation

Intracellular oxidation in sperm stored for 7 days was assessed using a DCFH-DA-based Reactive Oxygen Species Assay Kit (S0033S; Beyotime Biotechnology, Shanghai, China), following the method described in our previous study [25]. Briefly, sperm samples were incubated with the fluorescent probe 2′,7′-dichlorodihydrofluorescein diacetate (DCFH-DA; final concentration, 10 μM) at 37 °C for 20 min in the dark. The samples were gently mixed at regular intervals during incubation to facilitate uniform probe loading. After staining, the sperm were washed with serum-free medium to remove excess extracellular probe and immediately analyzed using a flow cytometer (BeamCyte; Changzhou, China). The fluorescence signal generated after intracellular probe oxidation was excited using a 488 nm laser and detected in the FITC channel. A total of 20,000 sperm events were acquired for each sample. After exclusion of debris and sperm aggregates, intracellular oxidation was quantified as the mean fluorescence intensity recorded in the gated sperm population. Flow cytometry data were processed using FlowJo software (version 10.8.1). The assay was performed using three independent pooled semen collections as biological replicates.

### 2.12. Apoptosis Detection

Sperm apoptosis after 7 days of storage was assessed using an Annexin V-FITC Apoptosis Detection Kit (C1062L; Beyotime Biotechnology, Shanghai, China), following the method described in our previous study [26], with minor modifications. Briefly, sperm samples were collected by centrifugation, and the sperm pellet was gently resuspended in 195 μL of Annexin V-FITC binding solution. Subsequently, 5 μL of Annexin V-FITC and 10 μL of propidium iodide (PI) staining solution were added. The samples were gently mixed and incubated at 25 °C for 10 min in the dark, followed by immediate analysis using a flow cytometer (BeamCyte; Changzhou, China). Appropriate unstained and single-stained controls were used to establish fluorescence compensation and quadrant boundaries. A total of 20,000 sperm events were acquired for each sample. Cellular debris and sperm aggregates were excluded before quadrant analysis. Annexin V-FITC^−^/PI^−^ sperm were classified as viable sperm; Annexin V-FITC^+^/PI^−^ sperm were classified as early apoptotic sperm; Annexin V-FITC^+^/PI^+^ sperm were classified as late apoptotic sperm or sperm with loss of membrane integrity; and Annexin V-FITC^−^/PI^+^ sperm were classified as membrane-compromised or necrotic sperm. Flow cytometry data were processed using FlowJo software (version 10.8.1). The assay was performed using three independent pooled semen collections as biological replicates. Individual flow cytometric events were not treated as independent biological replicates.

### 2.13. Thermo-Resistance Test

The thermo-resistance test (TRT) was performed as described in our previous study [27]. Briefly, semen samples stored for 7 days were incubated at 38 °C for up to 360 min, and sperm motility parameters were evaluated by CASA at 60 min intervals. TRT data were obtained from three independent pooled semen collections. Measurements from different CASA fields at each incubation time were averaged within each biological replicate before statistical analysis.

### 2.14. Capacitation

Sperm samples after 7 days of preservation were incubated with capacitation medium at 37 °C in a humidified incubator with 5% CO_2_ for 4 h to induce capacitation. Sperm capacitation status was assessed by chlortetracycline (CTC) staining according to Fraser et al. [28]. Briefly, the sperm samples were stained with 100 μL CTC solution at 37 °C for 20 min, then fixed with 5 μL 12.5% glutaraldehyde in 20 mM Tris-HCl, and evaluated with a fluorescence microscope (ZEISS DM200LED, Oberkochen, Germany). The capacitation assay was performed using three independent pooled semen collections as biological replicates. Microscopic fields were treated as technical measurements and were averaged within each biological replicate before statistical analysis.

The capacitation medium solution was prepared as follows:11 mM D-glucose, 5 mM sodium pyruvate, 113.1 mM sodium chloride, 3.13 mM Potassium Chloride, 7.5 mM Calcium chloride dihydrate, 19.84 mM Tris, 15 μM Bovine Serum Albumin.

### 2.15. Evaluation of the Ability of Sperm to Bind to the Oviduct Explants

As described in a previous study [29], the in vitro oviduct-binding ability of sperm was evaluated using oviduct explants. Oviductal tissues were collected from healthy sows. Explants were prepared from the oviductal isthmus and subsequently maintained in Tyrode’s medium. Only explants with intact ciliated structures were used for experiments. Hoechst 33342-stained sperm after 7 days of storage were incubated for 30 min. Explants were placed individually into pre-warmed Tyrode’s medium (5% CO_2_, 38 °C, 100% humidity) in 24-well plates and co-incubated with sperm (2 × 10^5^) for 45 min. After washing to remove loosely attached sperm, explants were mounted on glass slides with Tyrode’s medium and sealed with a coverslip. To minimize the edge effects from tissue preparation, the outermost layer of the explant was excluded, and three regions for analysis were randomly chosen within the remaining explant surface area. Images of different focal planes were combined to quantify sperm binding. Explant surface area and sperm count were determined to evaluate binding density. The average sperm bound per mm2 was defined as the binding index (BI). BI was determined using the following formula: BI = NI/AI, where AI represents the areas of three different regions, while NI represent the number of sperm bound to each of these regions. The oviduct explant binding assay was performed using sperm samples from three independent pooled semen collections as biological replicates. Multiple regions within each explant were treated as technical measurements and were averaged within each biological replicate before statistical analysis.

The formula of Tyrode’s medium is as follows: 100.5 mM NaCl, 20 mM HEPES, 15 mM NaHCO_3_, 5 mM glucose, 3.1 mM KCl, 2 mM CaCl_2_, 0.3 mM KH_2_PO_4_, 0.4 mM MgSO_4_, 21.7 mM sodium lactate, 1 mM sodium pyruvate, 100 μg/mL gentamicin sulphate, 20 μg/mL phenol red and 3 mg/mL BSA (pH 7.6 ± 0.05).

### 2.16. Western Blotting

Western blotting was performed as described previously [27]. After 7 days of storage, sperm samples were incubated under capacitating conditions for 4 h and lysed by sonication in radioimmunoprecipitation assay (RIPA) buffer supplemented with protease inhibitors. The lysates were centrifuged at 12,000× *g* for 15 min, and the supernatants were collected. Protein extracts were mixed with SDS sample loading buffer, boiled for 10 min, separated on 10% SDS-polyacrylamide gels, and transferred onto polyvinylidene fluoride membranes. The membranes were blocked with 5% bovine serum albumin in TBST and incubated with an anti-phosphotyrosine antibody (Abcam, Shanghai, China; Cat. No. ab179530), followed by an HRP-conjugated secondary antibody (ABclonal, Wuhan, China; Cat. No. AS014). Protein signals were detected using an enhanced chemiluminescence reagent. The membranes were subsequently incubated with an anti-α-tubulin antibody (ABclonal, Wuhan, China; Cat. No. AC007), which was used as the loading control. Phosphotyrosine signals were quantified and normalized to α-tubulin. Western blotting was performed using samples from three independent pooled semen collections.

### 2.17. Data Analysis

Statistical analyses were conducted using IBM SPSS Statistics 26.0 (IBM Corp., Armonk, NY, USA). The independent pooled semen collection was considered the experimental unit. CASA fields, flow cytometric events, repeated wells, and image fields were treated as technical measurements and were averaged within each biological replicate before statistical analysis. Data are presented as mean ± standard error of the mean (SEM) from three independent pooled semen collections.

For sperm motility and kinematic parameters, comparisons among treatments were performed using one-way analysis of variance (ANOVA) at each storage time point. Only data from the same storage time were compared, and no comparisons were made across different storage times. When significant differences were detected, multiple comparisons among treatments within the same time point were conducted using Tukey’s post hoc test. In addition to formal tests, ANOVA assumptions were further assessed graphically using residual Q–Q plots and residual-versus-fitted plots.

For variables measured only after 7 days of storage, treatment was included as a fixed effect. For comparisons between the control and 100 μM L-His groups derived from the same pooled semen collections, paired comparisons were used. Data were first tested for normality using the Shapiro–Wilk test and for homogeneity of variance using Levene’s test. Percentage data were transformed using the arcsine square-root method when required. Statistical significance was set at *p* < 0.05. Graphs were generated using GraphPad Prism 8.0 (GraphPad Software, San Diego, CA, USA).

## 3. Results

### 3.1. L-His Improves Boar Sperm Motility, Acrosomal Integrity and Viability

**Figure 1:**
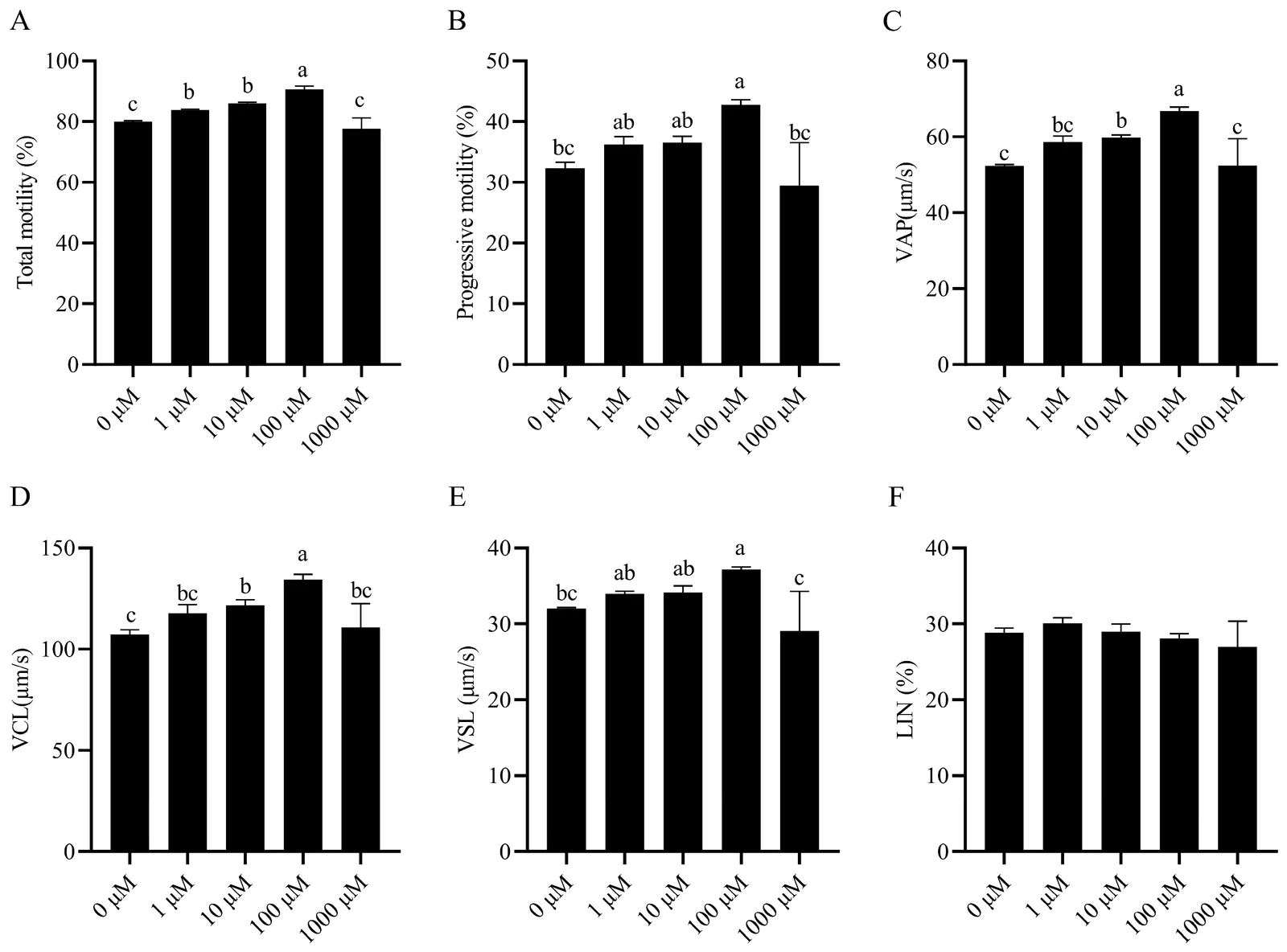
Effects of L-His supplementation on boar sperm motility and kinematic parameters after 7 days of liquid storage at 17 °C. (**A**) Total motility (TM); (**B**) progressive motility (PM); (**C**) average path velocity (VAP); (**D**) curvilinear velocity (VCL); (**E**) straight-line velocity (VSL); and (**F**) linearity (LIN). Data are presented as mean ± SEM (*n* = 3). Different lowercase letters indicate significant differences among treatment groups (*p* < 0.05). The absence of letters indicates no significant difference among groups.

Sperm motility and kinematic parameters were evaluated on days 1, 3, 5, and 7 of storage. No significant differences were observed among the treatment groups on days 1 and 3 (Table S1). On day 5, supplementation with 1, 10, or 100 μM L-His resulted in significantly higher TM than the control (*p* < 0.05), with no significant differences among these three concentrations. The remaining CASA parameters did not differ significantly between the control and the 1–100 μM L-His groups, whereas 1000 μM L-His did not provide a comparable benefit (Table S1). After 7 days of storage, the most pronounced improvement was observed with 100 μM L-His. This treatment resulted in significantly higher TM, VAP, and VCL than all other groups (*p* < 0.05; Figure 1A,C,D). PM and VSL were also significantly higher than those in the control and 1000 μM groups but did not differ significantly from the 1 and 10 μM groups (*p* < 0.05; Figure 1B,E). Supplementation with 1 or 10 μM L-His also increased TM, while 10 μM L-His increased VAP and VCL compared with the control (*p* < 0.05). In contrast, 1000 μM L-His did not improve the measured motility parameters, and LIN did not differ significantly among the groups (*p* > 0.05; Figure 1F).

**Figure 2:**
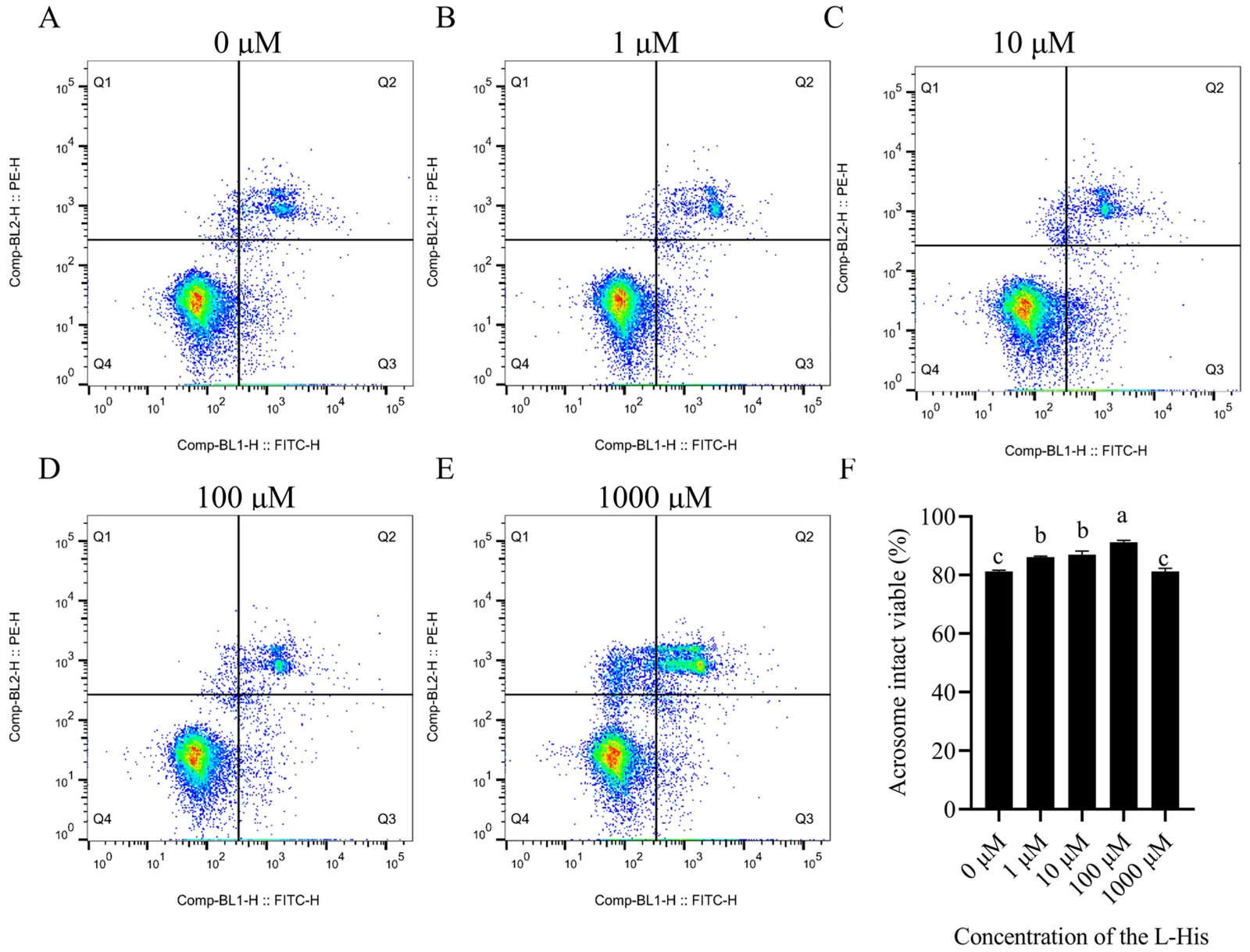
Effect of L-His supplementation on acrosomal integrity and viability of boar sperm after 7 days of liquid storage at 17 °C. (**A**–**E**) Flow cytometric analysis of acrosome integrity and viability in boar sperm. Q1 (FITC-PNA^−^/PI^+^), dead sperm with intact acrosomes; Q2 (FITC-PNA^+^/PI^+^), dead sperm with damaged acrosomes; Q3 (FITC-PNA^+^/PI^−^), live sperm with damaged acrosomes; and Q4 (FITC-PNA^−^/PI^−^), live sperm with intact acrosomes. (**F**) The percentage of sperm with viability and acrosome integrity. Statistical analysis was performed using one-way ANOVA followed by Tukey’s post hoc test. Data are presented as mean ± SEM from three independent pooled semen collections (*n* = 3). Different lowercase letters in each column indicate statistically significant differences (*p* < 0.05).

With respect to sperm viability and acrosomal integrity, no significant differences were observed among the treatment groups on days 1 and 3 of storage, and sperm in all groups maintained high proportions of acrosome-intact viable sperm (Figure S1A,B). On day 5, supplementation with 10 μM and 100 μM L-His significantly increased the percentage of acrosome-intact viable sperm compared with the control group (Figure S1C, *p* < 0.05). However, no significant differences were detected between the 10 μM and 100 μM L-His groups. After 7 days of storage, supplementation with L-His at concentrations ranging from 1 to 100 μM significantly increased the proportion of acrosome-intact viable sperm compared with the control group, with the 100 μM L-His group showing the highest value among all treatments, significantly higher than the other groups (Figure 2F, *p* < 0.05). In contrast, supplementation with 1000 μM L-His did not significantly alter the percentage of acrosome-intact viable sperm compared with the control group (*p* > 0.05). Based on these results, subsequent experiments were conducted using sperm stored for 7 days.

### 3.2. L-His Enhanced Boar Sperm Mitochondrial Membrane Potential and ATP Levels After 7 Days of Storage at 17 °C

**Figure 3:**
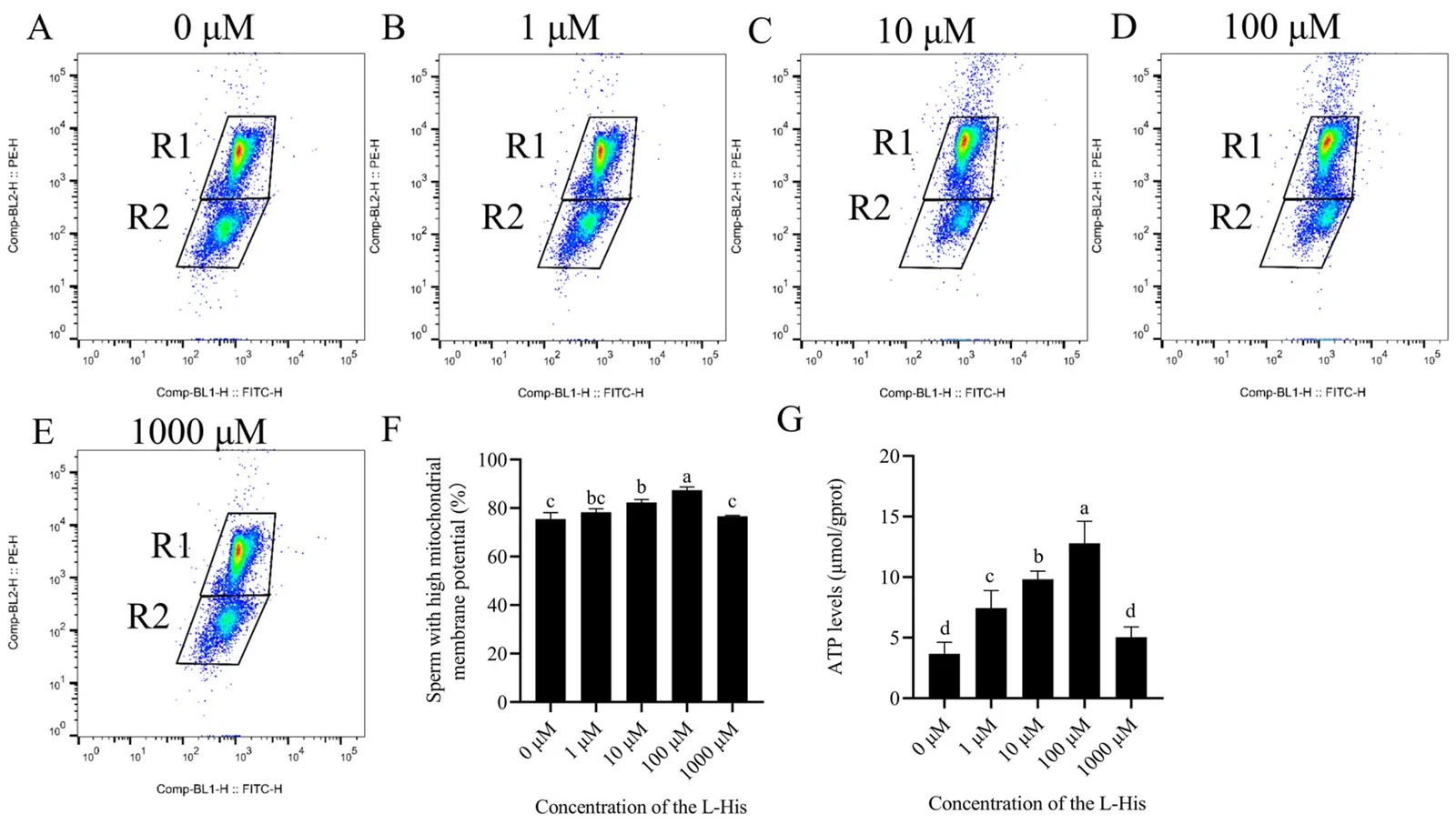
The effect of L-His on sperm mitochondrial membrane potential (MMP) and ATP levels after 7 days of preservation at 17 °C. (**A**–**E**) Flow cytometry analysis of sperm MMP. The R1 region refers to sperm with high mitochondrial membrane potential, while the R2 region refers to sperm with low mitochondrial membrane potential. (**F**) Sperm with high mitochondrial membrane potential; (**G**) ATP levels. Statistical analysis was performed using one-way ANOVA followed by Tukey’s post hoc test. Data are presented as mean ± SEM from three independent pooled semen collections (*n* = 3). Different lowercase letters above the bars indicate statistically significant differences among treatment groups (*p* < 0.05).

Supplementation with 10 and 100 μM L-His significantly increased the proportion of sperm with high mitochondrial membrane potential (MMP) compared with the control group (*p* < 0.05), with the 100 μM group exhibiting the highest value (Figure 3A–F). In contrast, MMP was significantly reduced in the 1000 μM L-His group compared with the 100 μM L-His group.

Similarly, compared to the control, the addition of L-His from 1 to 100 μM significantly enhanced ATP levels after 7 days of storage at 17 °C (*p* < 0.05), showing a trend consistent with the MMP results (Figure 3G).

### 3.3. L-His Reduced Intracellular Oxidation and Lipid Peroxidation During Storage at 17 °C

**Figure 4:**
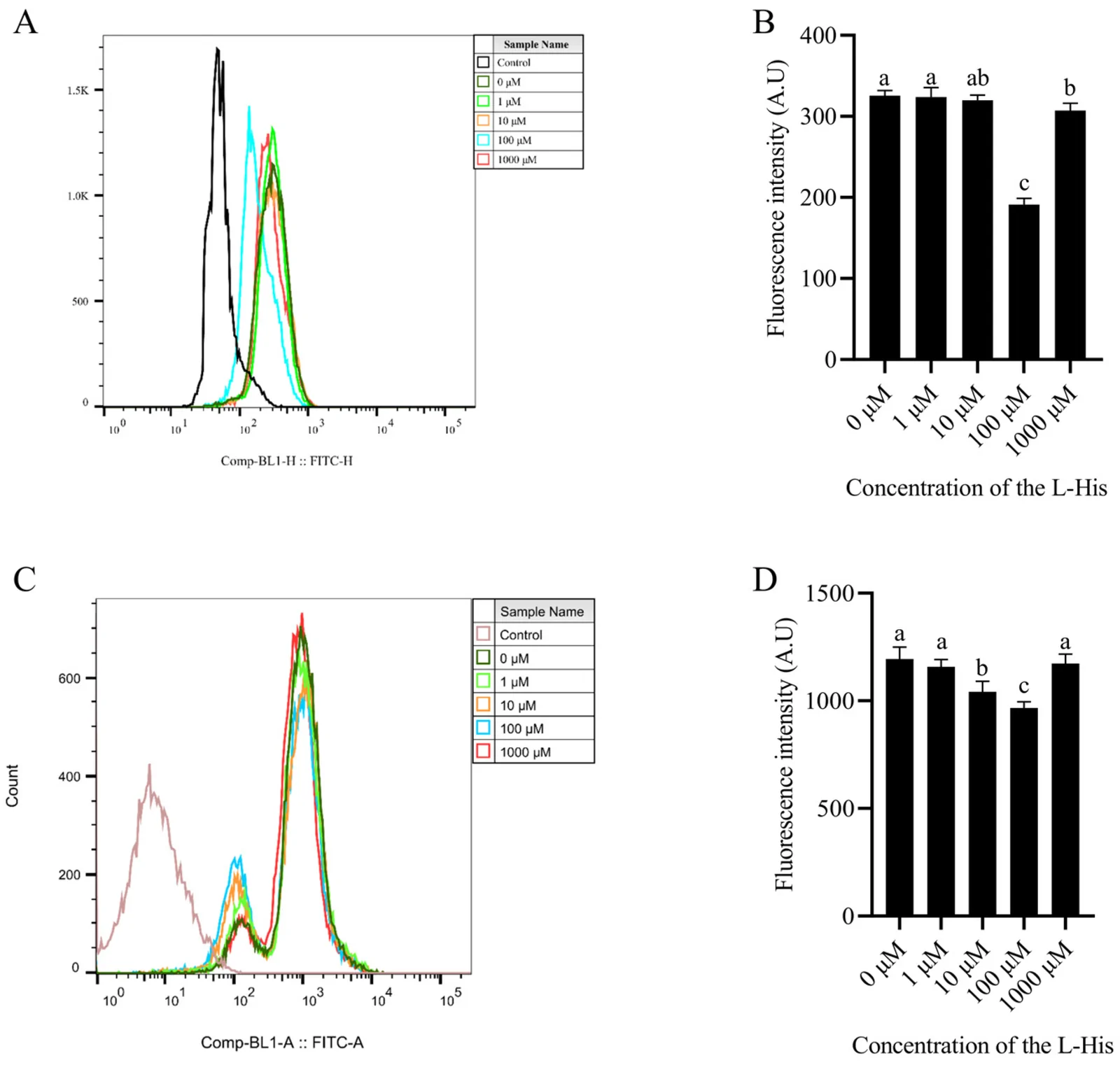
Effects of L-His on lipid peroxidation and intracellular oxidation in boar sperm after 7 days of preservation at 17 °C. (**A**,**B**) Flow cytometric assessment of lipid peroxidation; (**C**,**D**) flow cytometric assessment of intracellular oxidation using DCFH-DA. Statistical analysis was performed using one-way ANOVA followed by Tukey’s post hoc test. Data are presented as mean ± SEM from three independent pooled semen collections (*n* = 3). Different lowercase letters in each column of the same image indicate statistically significant differences (*p* < 0.05).

Supplementation with 100 and 1000 μM L-His significantly decreased the sperm LPO level compared to the control after 7 days of liquid storage at 17 °C (Figure 4A,B, *p* < 0.05). In addition, intracellular oxidation was significantly lower in the 10 and 100 μM L-His groups than in the control group (*p* < 0.05), with the lowest value observed in the 100 μM L-His group, whereas the 1 and 1000 μM groups did not differ significantly from the control (Figure 4C,D).

### 3.4. L-His Reduced Boar Sperm Apoptosis After 7 Days of Storage at 17 °C

**Figure 5:**
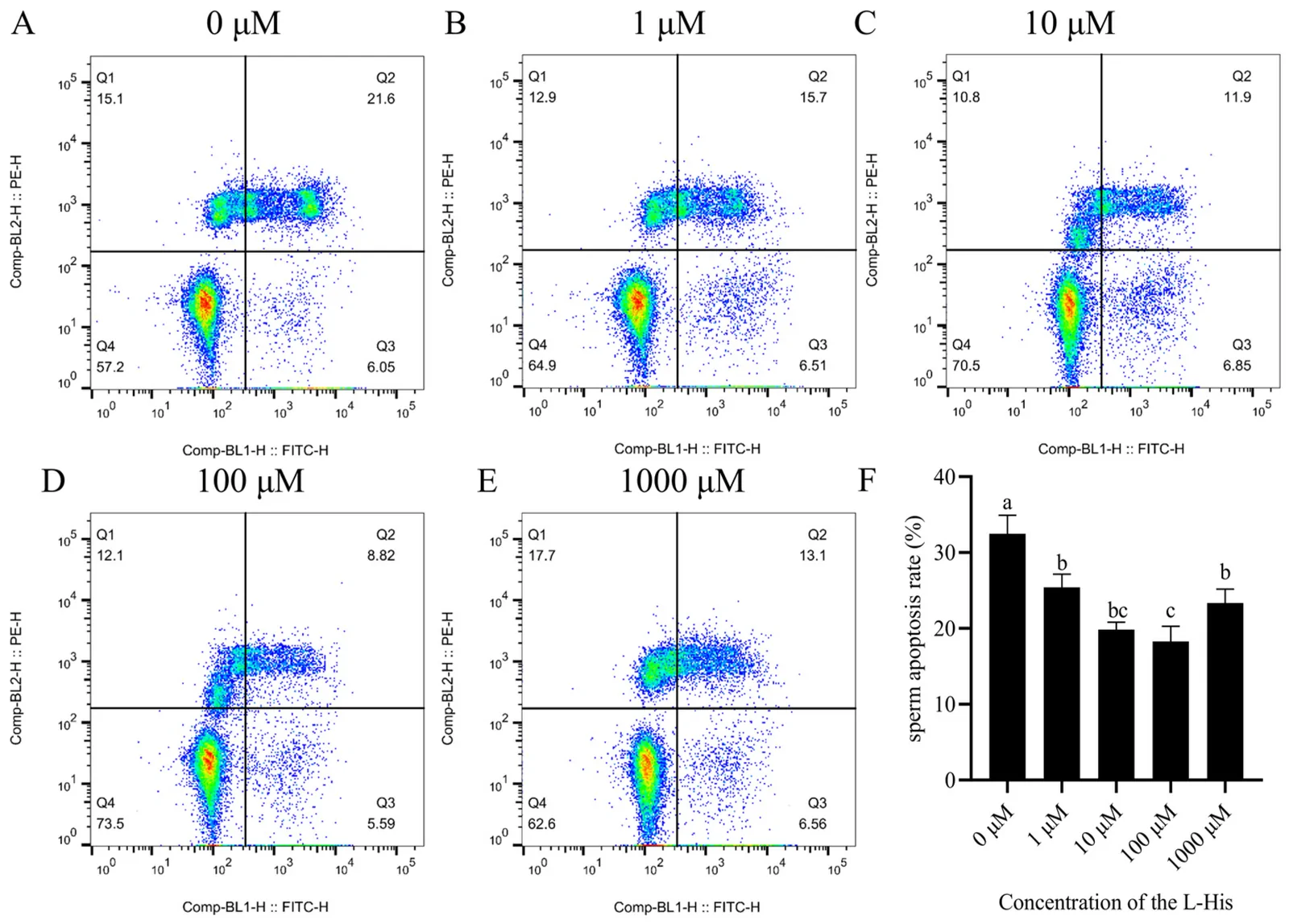
The effect of L-His on sperm apoptosis after 7 days of preservation at 17 °C. (**A**–**F**) Flow cytometry analysis of sperm apoptosis. Q1 represents dead sperm. Q2 represents late apoptotic sperm; Q3 represents early apoptotic sperm. Q4 represents viable sperm. Statistical analysis was performed using one-way ANOVA followed by Tukey’s post hoc test. Data are presented as mean ± SEM from three independent pooled semen collections (*n* = 3). Different lowercase letters represent significant differences (*p* < 0.05).

Sperm apoptosis in each treatment after 7 days of preservation was evaluated with an Annexin V-FITC/PI kit. As shown in Figure 5A–F, L-His supplementation significantly reduced sperm apoptosis (*p* < 0.05). In particular, the group treated with 100 μM L-His displayed a significantly lower proportion of apoptotic sperm when compared with the control group (*p* < 0.05).

### 3.5. L-His Enhanced Sperm Thermo-Resistance After 7 Days of Storage

**Figure 6:**
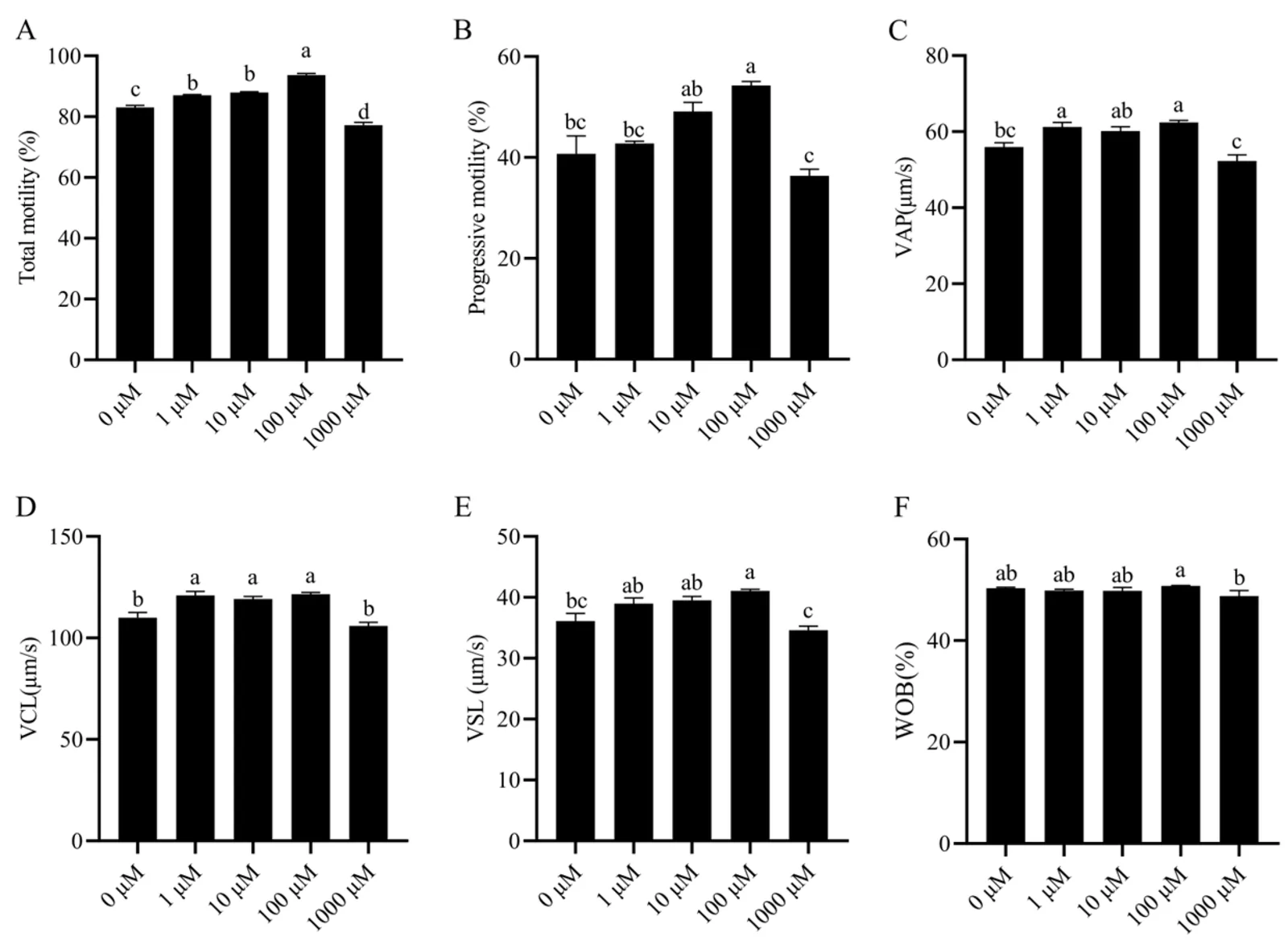
Effects of L-His supplementation on boar sperm motility and kinematic parameters after 3 h of thermo-resistance testing. Semen samples stored at 17 °C for 7 days were incubated at 38 °C for 3 h, after which (**A**) total motility (TM), (**B**) progressive motility (PM), (**C**) average path velocity (VAP), (**D**) curvilinear velocity (VCL), (**E**) straight-line velocity (VSL), and (**F**) wobble (WOB) were evaluated. Data are presented as mean ± SEM (*n* = 3). Different lowercase letters indicate significant differences among treatment groups (*p* < 0.05). The absence of letters indicates no significant difference among groups.

The TRT was performed to evaluate whether L-His supplementation improved the resistance of stored boar sperm to prolonged incubation at 38 °C. Throughout the 1–6 h incubation period, the 100 μM L-His group consistently showed the highest mean TM and generally higher PM, VAP, VCL, VSL, and ALH than the control, although statistical significance varied among incubation times and CASA parameters (Figure 6 and Table S2). After 1 h, TM and PM were significantly higher in the 100 μM L-His group than in the control group (*p* < 0.05). After 3 h, supplementation with 100 μM L-His resulted in significantly higher TM, PM, VAP, VCL, VSL, and ALH than the control group (*p* < 0.05), whereas WOB did not differ significantly between the two groups (Figure 6 and Table S2). The protective effect remained evident after 6 h, when the 100 μM L-His group maintained significantly higher TM and PM than the control group (*p* < 0.05). Lower L-His concentrations produced smaller or time-dependent effects, whereas 1000 μM L-His did not provide comparable protection and generally resulted in lower TM and PM than 100 μM L-His during the later stages of the TRT.

### 3.6. L-His Maintained in Vitro Capacitation-Related Sperm Function During Storage at 17 °C

**Figure 7:**
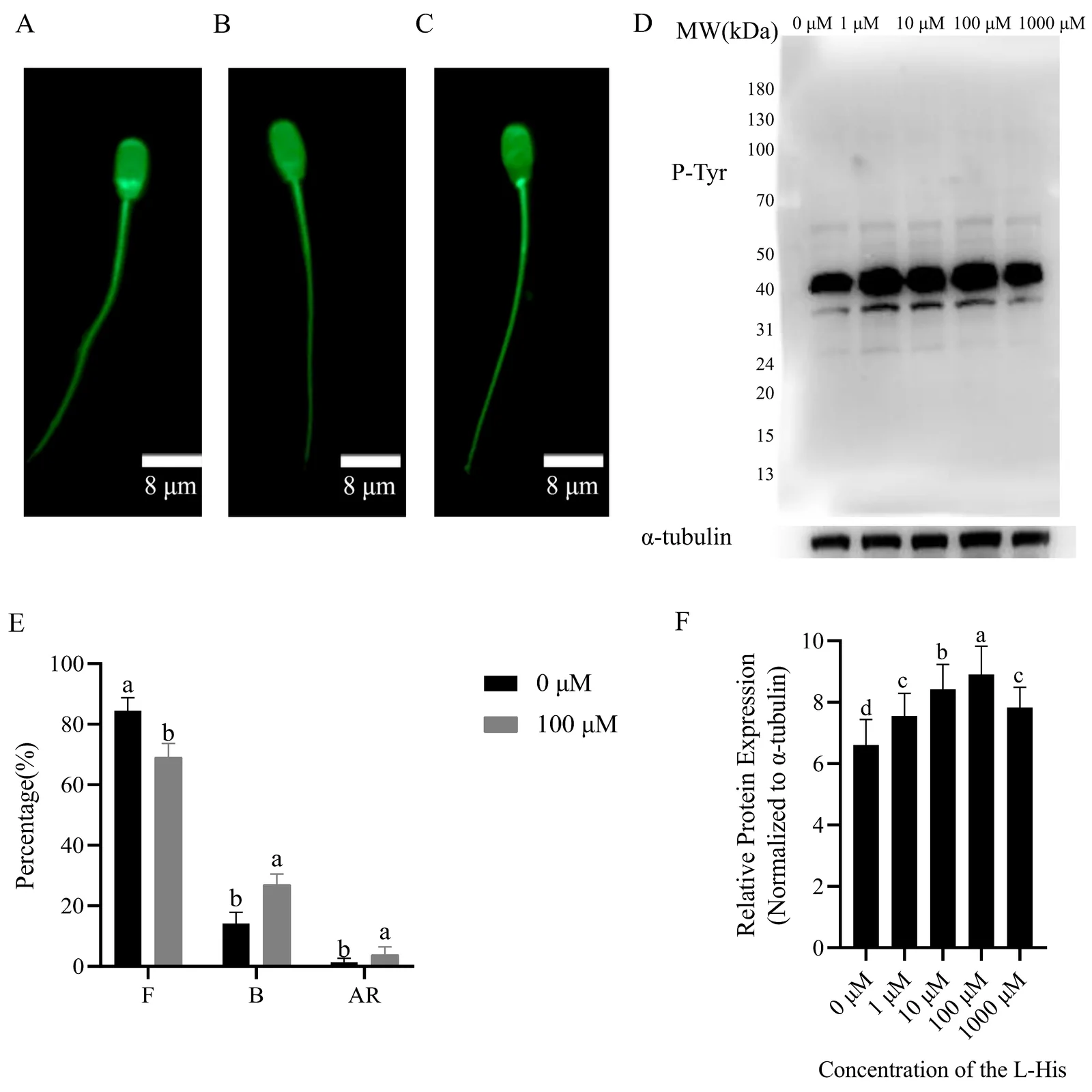
Effect of L-His on capacitation-related parameters of boar sperm after 7 days of storage. (**A**–**C**) Representative chlortetracycline (CTC) staining patterns: (**A**) AR pattern, acrosome-reacted sperm showing dull fluorescence over the sperm head, except for a thin, bright band in the equatorial segment; (**B**) F pattern, uncapacitated sperm showing uniform fluorescence over the entire sperm head; and (**C**) B pattern, capacitated sperm showing a fluorescence-free band in the post-acrosomal region. Scale bars = 8 μm. (**D**) Representative immunoblot of tyrosine-phosphorylated proteins, with α-tubulin used as the reference protein. Molecular mass markers are indicated in kDa. (**E**) Percentages of uncapacitated (F), capacitated (B), and acrosome-reacted (AR) sperm following capacitation induction. (**F**) Relative tyrosine phosphorylation levels, quantified as the ratio of the total signal intensity of tyrosine-phosphorylated proteins to that of α-tubulin. Data are presented as mean ± SEM from three independent pooled semen collections (*n* = 3). Differences among treatments were assessed using one-way ANOVA followed by Tukey’s multiple-comparison test. Bars bearing different lowercase letters differ significantly (*p* < 0.05).

To further evaluate the effect of L-His on boar sperm function after preservation, sperm capacitation ability was evaluated. As shown in Figure 7A–C, three CTC fluorescence patterns were observed: acrosome-reacted sperm (AR), characterized by weak fluorescence over the whole head except for a thin, bright band in the equatorial segment, uncapacitated sperm (F), characterized by uniform fluorescence across the entire sperm head; and capacitated sperm (B), characterized by a fluorescence-free band in the post-acrosomal region. Following the induction of capacitation, sperm stored for 7 days in the presence of 100 μM L-His exhibited higher proportions of capacitated and acrosome-reacted sperm than the control group (*p* < 0.05; Figure 7E). In addition, the relative protein tyrosine phosphorylation level was significantly higher in the 100 μM L-His group than in the control group under capacitating conditions (*p* < 0.05; Figure 7D,F).

### 3.7. L-His Improves the Binding Capacity of Sperm to Oviduct Explants

**Figure 8:**
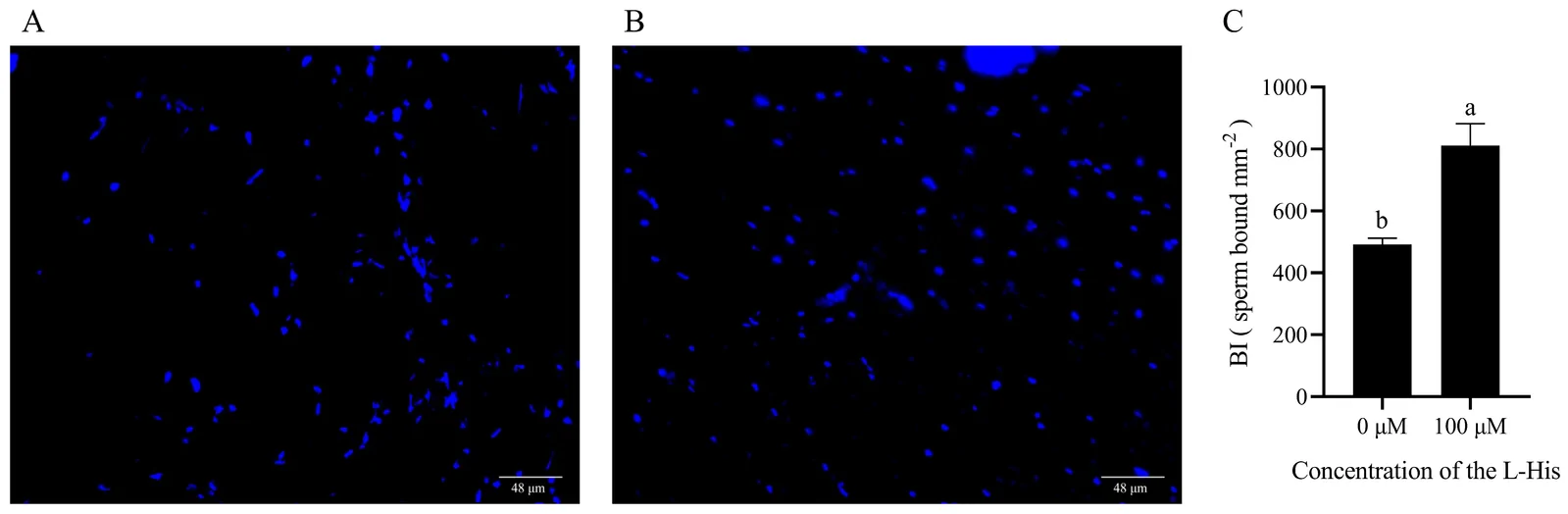
Effect of L-His supplementation on the oviduct-binding ability of boar sperm after 7 days of liquid storage at 17 °C. (**A**) Control group; (**B**) 100 μM L-His group. Representative images showing sperm bound to the oviductal epithelial surface after 7 days of liquid storage (scale bars = 48 μm); (**C**) sperm binding index (BI). Statistical analysis was performed using a paired *t*-test. Data are presented as mean ± SEM from three independent pooled semen collections (*n* = 3). Different lowercase letters represent significant differences (*p* < 0.05).

The effect of L-His supplementation on the in vitro oviduct-binding ability of boar sperm after 7 days of storage at 17 °C was evaluated. Sperm–oviduct binding was assessed using the binding index (BI), with bound sperm visualized by Hoechst 33342 staining. As shown in Figure 8, sperm BI in the 100 μM L-His group was significantly higher than that in the control group (*p* < 0.05), suggesting that L-His supplementation may enhance the reservoir-forming potential of stored sperm.

## 4. Discussion

ROS participate in sperm motility, capacitation, and fertilization, whereas excessive ROS accumulation during semen storage can cause oxidative damage [30]. Boar sperm are particularly susceptible to such damage because their plasma membranes are rich in polyunsaturated fatty acids and their antioxidant defenses are relatively limited [4,5]. Excess ROS can consequently initiate lipid peroxidation and impair both membrane and mitochondrial function [31,32]. Nevertheless, oxidation of polyunsaturated fatty acids is not invariably harmful. Controlled oxidative pathways also generate bioactive lipid mediators, including prostaglandin E2 (PGE2), that contribute to the regulation of sperm function [33]. After 7 days of storage, sperm supplemented with 100 μM L-His had lower intracellular probe oxidation and membrane lipid oxidation, while maintaining higher motility and a greater proportion of viable, acrosome-intact cells. Interpretation of these findings requires consideration of what the two probes measure. DCFH-DA provides an overall indication of intracellular probe oxidation but does not distinguish among individual ROS species. For the BODIPY measurement, the stored samples were washed before the probe was added. The fluorescence recorded after the 30 min incubation concerns membrane lipid oxidation during exposure to BODIPY 581/591 C11. It does not quantify 4-HNE or MDA that may have accumulated earlier during storage.

Histidine may also provide protection through reactions with lipid-derived electrophilic aldehydes rather than through strong direct radical scavenging. Among these aldehydes, 4-hydroxynonenal (4-HNE) can modify sperm proteins and compromise their function [34]. Although histidine reacts relatively weakly with several reactive species [14], its imidazole group can form covalent adducts with lipid-derived electrophilic aldehydes. This chemical property has previously been considered in the development of long-term preservation media for human sperm [15]. Histidine has also been shown to form adducts with 4-HNE and reduce 4-HNE-associated cellular injury [35]. The lower membrane lipid oxidation and better preservation of sperm function observed with 100 μM L-His are consistent with such a mechanism. However, neither 4-HNE nor histidine–aldehyde adducts were measured in this study, and the involvement of aldehyde trapping remains unconfirmed.

The concentration–response relationship was not linear. Supplementation with 1–100 μM L-His generally improved sperm quality, with the clearest overall response occurring at 100 μM, whereas most of these benefits were absent at 1000 μM. The composition of sperm preservation media can itself influence sperm survival and functional quality [15], and similar concentration-dependent responses have been reported for other additives used in boar semen. Carvacrol improved several sperm characteristics at relatively low concentrations but became less effective or detrimental as its concentration increased [36]. MitoQ also showed an optimal concentration during boar sperm cryopreservation, whereas excessive supplementation reduced sperm quality and antioxidant status [37]. Such responses may partly reflect the physiological requirement for controlled ROS signaling. ROS participate in capacitation-associated events and redox-sensitive protein phosphorylation [38,39], meaning that greater antioxidant activity does not necessarily translate into better sperm function. In the 1000 μM L-His group, however, intracellular probe oxidation was similar to that in the control group, while membrane lipid oxidation remained relatively low. The poorer sperm quality at this concentration therefore cannot be explained simply by increased oxidative damage. Other concentration-dependent effects of L-His on the extender environment or sperm cellular homeostasis require further investigation.

Sperm motility depends on a continuous ATP supply generated through glycolysis and mitochondrial oxidative phosphorylation [40]. Mitochondrial dysfunction is closely associated with oxidative stress and impaired sperm function, and mitochondrial activity makes an important contribution to energy metabolism in boar sperm [41,42,43]. Cellular ATP availability is also closely related to the maintenance of sperm motility [44]. On day 7, the higher MMP and ATP levels in sperm stored with 100 μM L-His were accompanied by higher motility. This pattern is consistent with better preservation of mitochondrial status and cellular energy availability, although it does not identify the metabolic source of the additional ATP. Aldehyde trapping may also be relevant to mitochondrial preservation. In human sperm, 4-HNE can form adducts with succinate dehydrogenase, impair its activity, and promote mitochondrial oxidant generation [45]. By limiting the exposure of mitochondrial proteins to lipid-derived aldehydes, L-His could therefore interrupt this cycle of mitochondrial damage and oxidant production, although this possibility was not directly tested. Sperm stored with 100 μM L-His also retained higher TM and PM during prolonged incubation in the thermo-resistance test, which is commonly used to assess the capacity of stored boar sperm to maintain motility under sustained incubation [46]. This functional response agreed with the higher MMP and better membrane status detected after storage. Nevertheless, ATP was measured as total cellular content, while mitochondrial respiration and glycolytic activity were not assessed separately. The higher ATP content cannot therefore be attributed specifically to increased mitochondrial oxidative phosphorylation.

Oxidative deterioration during prolonged semen storage can be accompanied by phosphatidylserine externalization and loss of plasma membrane integrity, which are commonly described as apoptotic changes in sperm [5]. Annexin V staining has accordingly been used in boar sperm to evaluate phosphatidylserine translocation together with membrane integrity [47]. Supplementation with 100 μM L-His reduced the proportions of Annexin V-positive and membrane-compromised sperm after 7 days. This pattern was consistent with the lower probe oxidation and the greater proportion of membrane-intact sperm in the same treatment. Annexin V-FITC/PI staining alone, however, does not demonstrate activation of a specific apoptotic pathway. 

Capacitation is required for sperm to acquire full fertilizing competence and involves coordinated changes in membrane properties and protein phosphorylation [48]. ROS contribute to capacitation-related signaling, including tyrosine phosphorylation, whereas excessive oxidative stress can disrupt these events [49]. After capacitation induction, sperm stored with 100 μM L-His showed higher proportions of capacitated and acrosome-reacted cells together with greater protein tyrosine phosphorylation than control sperm. The timing of these measurements is important: they were obtained after exposure to a defined capacitation protocol. The results therefore indicate that L-His-treated sperm retained greater responsiveness to capacitating conditions, rather than showing that L-His induced capacitation during storage. Because ROS were not measured during capacitation, the stronger response cannot be attributed directly to altered redox signaling.

Sperm binding to the porcine oviductal epithelium contributes to the formation of the sperm reservoir, which maintains a population of functionally competent sperm before fertilization [50,51]. Following 7 days of storage, sperm preserved with 100 μM L-His had a higher binding index to oviductal explants than control sperm, indicating better retention of cellular properties involved in sperm–oviduct interaction. Nevertheless, this in vitro assay evaluates binding capacity and does not necessarily predict fertility after artificial insemination [52]. By examining post-storage capacitation responses and oviductal binding alongside conventional sperm-quality measurements, the present study broadens the limited functional evidence available for L-His in sperm preservation. Whether these benefits improve reproductive performance in vivo remains to be established. Future work could also determine whether combining L-His with ergothioneine provides complementary protection during prolonged boar semen storage.

## 5. Conclusions

At 100 μM, L-His helped maintain boar sperm quality during prolonged liquid storage at 17 °C. The treatment was associated with lower intracellular probe oxidation and membrane lipid oxidation, higher MMP and ATP levels, and a greater proportion of membrane-intact sperm. Sperm stored with L-His also responded better to capacitating conditions and retained a greater capacity to bind to oviductal explants in vitro. By evaluating both preservation quality and post-storage functional responses, this study expands the limited evidence concerning the use of L-His in sperm preservation and supports its further evaluation as a boar semen extender supplement. Whether these in vitro benefits improve reproductive performance after artificial insemination remains to be tested.

## Data Availability

The raw data supporting the conclusions of this article will be made available by the corresponding authors upon reasonable request.

## Supplementary Materials

The following supporting information can be downloaded at: https://www.mdpi.com/article/10.3390/antiox15091086/s1, Figure S1: Effects of L-His supplementation on the proportion of viable sperm with intact acrosomes during liquid storage at 17 °C. Table S1. The effect of L-His supplementation in the semen extender on sperm motility parameters during storage at 17 °C. Table S2. Effect of L-His Supplementation on the Thermo-Resistance of Boar Sperm After 7 Days of Liquid Storage at 17 °C.

## Abbreviations

The following abbreviations are used in this manuscript:

AI	artificial insemination
ALH	amplitude of lateral head displacement
ATP	adenosine triphosphate
BCF	beat-cross frequency
BI	binding index
CASA	computer-assisted sperm analysis
CTC	chlortetracycline
L-His	L-histidine
LIN	linearity
LPO	lipid peroxidation
MMP	mitochondrial membrane potential
PM	progressive motility
ROS	reactive oxygen species
STR	straightness
TM	total motility
TRT	thermo-resistance test
VAP	average path velocity
VCL	curvilinear velocity
VSL	straight-line velocity
WOB	wobble
