# Supplementary material for: L-Histidine Improves Boar Sperm Quality by Alleviating Oxidative Stress During Preservation at 17 °C
Source: Antioxidants (Basel). 2026 Aug 28;15(9):1086. doi: 10.3390/antiox15091086 (PMC13603103; doi:10.3390/antiox15091086)
Supplement: Supplementary file 1 [file antioxidants-15-01086-s001.zip › antioxidants-4494620-supplementary.pdf]

Supplementary Materials

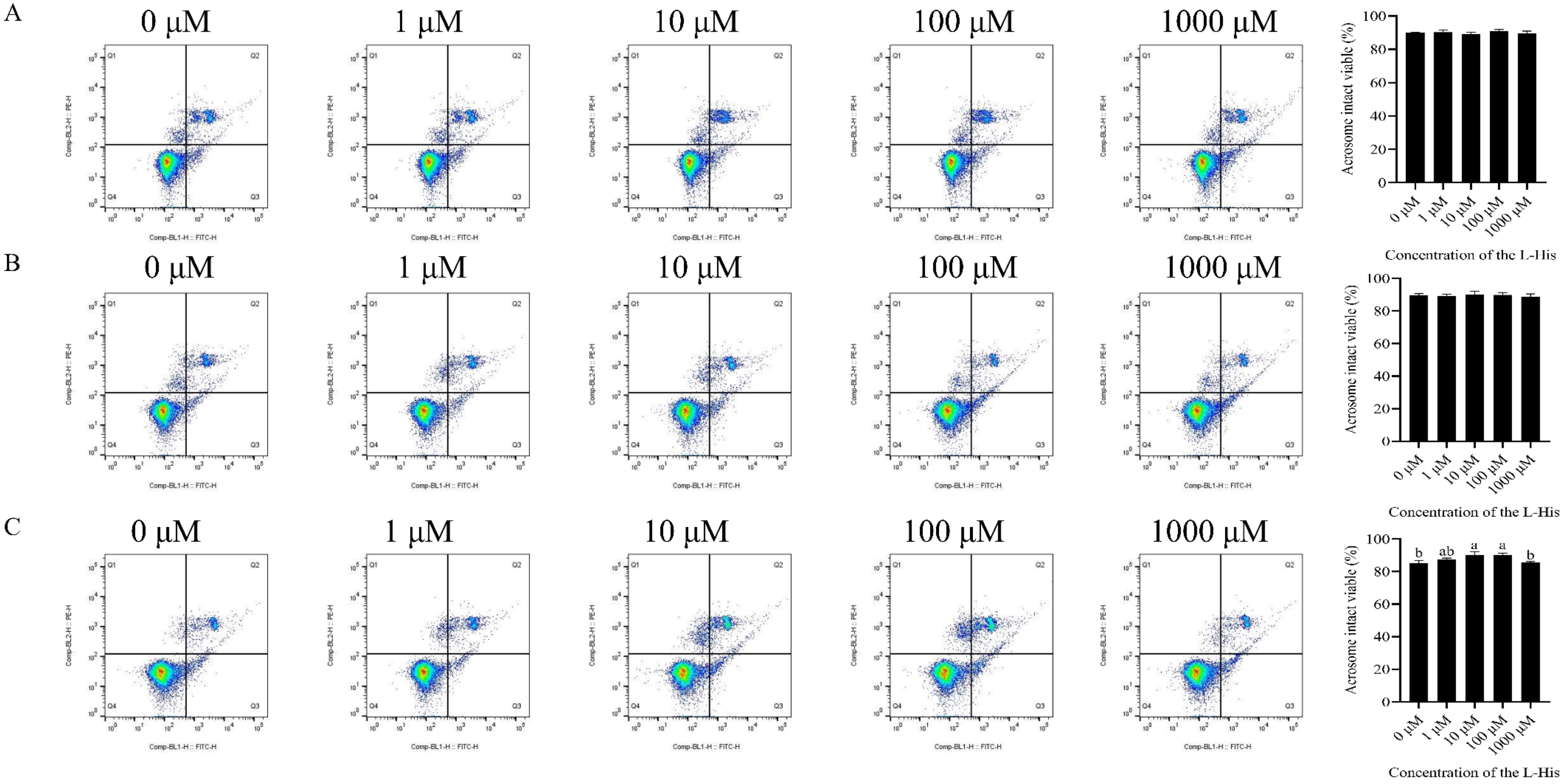

Figure S1 Effects of L-His supplementation on the proportion of viable sperm with intact acrosomes during liquid storage at 17 °C. Representative flow cytometric plots and percentages of viable sperm with intact acrosomes after (A) 1 day, (B) 3 days, and (C) 5 days of storage. Q1 (FITC-PNA−/PI+), non-viable sperm with intact acrosomes; Q2 (FITC-PNA+/PI+), non-viable sperm with damaged acrosomes; Q3 (FITC-PNA+/PI−), viable sperm with damaged acrosomes; and Q4 (FITC-PNA−/PI−), viable sperm with intact acrosomes. Data are presented as the mean ± SEM from three independent pooled semen collections. Statistical analysis was performed using one-way ANOVA followed by Tukey's post hoc test. Different lowercase letters indicate significant differences among treatments at the same storage time ($P < 0.05$). No significant differences were detected among treatments on days 1 and 3.

Table S1. The effect of L-His supplementation in the semen extender on sperm motility parameters during storage at 17 °c.

Parameters	Time (d)	0 μM	1 μM	10 μM	100 Mm	1000 μM
Total motility (%)	1	97.5±0.7	97.9±0.6	97.8±0.9	97.8±0.6	98.4±0.4
	3	94.5±0.5	94.2±0.6	94.1±0.6	94.8±0.4	93.7±0.6
	5	89.6±0.2b	92.2±0.9a	94.0±0.5a	94.4±1.0a	84.0±1.0c
	7	79.9±0.4c	83.8±0.3b	86.0±0.3b	90.7±1.0a	77.6±2.1c
Progressive motility (%)	1	47.0±1.1	47.8±0.7	48.3±0.9	47.3±1.6	48.6±0.5
	3	46.4±0.2	46.7±0.6	48.3±1.8	46.9±5.2	46.7±1.5
	5	41.4±2.5ab	41.2±1.2ab	41.7±2.3ab	43.8±2.3a	32.9±5.4b
	7	32.3±1.0bc	36.2±1.3ab	36.5±1.1ab	42.8±0.8a	29.4±4.1bc
VCL (μm/s)	1	173.8±3.7	164.8±2.7	169.7±7.9	171.1±8.9	169.5±5.4
	3	162.7±3.3	166.6±6.7	165.0±8.9	168.6±4.2	166.6±7.3
	5	148.5±1.2	154.3±3.1	170.0±3.1	158.8±6.4	159.4±13.1
	7	107.4±2.2c	117.7±4.3bc	121.7±2.7b	134.4±2.5a	110.9±6.7bc
VSL (μm/s)	1	43.1±1.1	42.2±1.1	43.2±2.2	43.3±1.8	43.1±1.0
	3	41.1±0.2	42.8±1.4	43.0±1.0	43.7±3.1	42.5±1.0
	5	37.5±1.8	38.6±1.6	40.4±0.9	40.2±1.5	35.7±1.7

	7	32.0±0.1bc	33.9±0.4ab	34.1±0.9ab	37.1±0.4a	29.1±3.0c
VAP (μm/s)	1	86.3±2.2	82.0±1.6	84.1±4.3	85.0±4.9	83.3±2.6
	3	80.6±1.5	82.9±3.0	81.9±3.9	83.7±1.9	82.7±2.7
	5	74.0±0.3	76.6±1.9	83.7±1.6	78.8±3.3	78.4±6.4
	7	52.4±0.3c	58.6±1.6bc	59.9±0.6b	66.8±1.0a	52.5±4.1c
STR (%)	1	49.4±0.2	50.7±0.6	51.4±0.2	50.5±1.0	51.4±0.7
	3	50.9±0.6	51.5±0.4	52.9±2.3	51.4±2.6	51.2±1.9
	5	51.0±1.8	50.3±0.8	48.1±1.0	50.8±0.5	46.6±5.9
	7	58.3±1.1	58.6±0.4	58.2±1.6	55.9±1.1	55.9±2.5
LIN (%)	1	25.1±0.2	25.7±0.3	26.0±0.2	25.7±0.5	25.6±0.5
	3	26.0±0.4	26.2±0.3	26.8±1.3	26.2±1.9	25.9±1.1
	5	26.3±1.2	25.4±0.5	24.2±0.6	25.7±0.1	23.5±3.0
	7	28.9±0.6	30.1±0.7	29.0±1.0	28.1±0.6	27.0±1.9
WOB (%)	1	49.4±0.3	49.5±0.1	49.6±0.3	49.6±0.3	49.0±0.1
	3	49.7±0.2	49.6±0.2	49.5±0.4	49.5±0.8	49.6±0.5
	5	49.9±0.5	49.2±0.3	49.1±0.3	49.3±0.3	48.9±0.2
	7	48.0±0.7	50.0±0.7	49.1±0.5	49.4±0.7	47.3±1.5
ALH (μm)	1	9.8±0.1	9.9±0.2	9.8±0.3	9.8±0.2	9.9±0.2
	3	9.6±0.1	9.6±0.2	9.4±0.4	9.6±0.1	9.4±0.4
	5	8.8±0.1	9.2±0.1	9.6±0.2	9.6±0.1	9.2±0.5
	7	8.2±0.1b	8.3±0.3ab	8.6±0.2ab	9.0±0.3a	7.4±0.1c
BCF (Hz)	1	35.5±0.2	35.3±0.2	35.0±0.3	35.5±0.0	36.0±0.4
	3	36.2±0.9	35.7±0.4	36.7±1.1	37.5±0.5	36.3±0.4
	5	36.0±0.4	35.4±0.4	36.2±0.3	35.2±0.6	36.4±1.1
	7	38.8±1.6	34.8±1.7	34.8±0.2	34.4±1.1	31.9±4.0

Data are presented as mean ± SEM from five independent pooled semen collections (n = 3). Six CASA fields were examined per sample and

averaged within each biological replicate before statistical analysis. Values within the same row bearing different superscript lowercase letters differ significantly among treatments at the same storage time ($p < 0.05$). VCL, curvilinear velocity; VSL, straight-line velocity; VAP, average path velocity; STR, straightness (VSL/VAP); LIN, linearity (VSL/VCL); WOB, wobble (VAP/VCL); ALH, amplitude of lateral head displacement; BCF, beat-cross frequency.

Table S2. Effect of L-His Supplementation on the Thermo-Resistance of Boar Sperm After 7 Days of Liquid Storage at 17 °C

Parameters	Time (h)	0 μM	1 μM	10 μM	100 μM	1000 μM
Total motility (%)	1	86.3±0.6b	86.6±0.7b	86.9±0.3b	90.5±0.1a	86.4±0.5b
	2	87.8±0.4bc	89.7±0.5ab	90.1±0.7ab	93.1±1.7a	83.4±0.8c
	3	83.0±0.7c	87.1±0.2b	87.9±0.3b	93.7±0.5a	77.2±0.5d
	4	79.0±2.4c	83.5±0.2bc	86.7±0.2b	93.5±0.6a	71.2±2.1d
	5	67.4±2.5c	77.5±0.6b	83.4±0.1ab	88.3±1.2a	66.9±1.3c
	6	58.5±1.8c	70.2±1.1b	80.7±0.7a	85.5±0.3a	57.1±0.8c
Progressive motility (%)	1	37.5±2.2b	43.1±1.2a	43.4±0.4a	46.5±0.4a	45.0±0.4a
	2	50.4±1.4a	50.3±0.6a	50.6±0.7a	53.8±2.2a	41.1±1.9b
	3	40.7±3.5bc	42.8±0.4bc	49.1±1.8ab	54.3±0.8a	36.4±0.7c
	4	38.3±3.8bc	41.2±2.0bc	42.7±1.2b	55.3±1.4a	32.0±0.3c
	5	33.6±3.1ab	37.2±2.6ab	39.5±2.7ab	45.9±2.7a	26.9±3.2b
	6	19.1±0.6c	29.1±3.1b	35.7±0.8ab	40.6±2.6a	19.7±0.6c
VCL (μm/s)	1	125.3±3.5b	126.0±2.4b	127.5±1.7b	152.4±1.6a	125.0±7.5b
	2	126.7±7.2ab	135.1±1.4a	138.9±1.0a	137.4±1.7a	113.6±3.8b
	3	109.9±2.7b	121.0±2.0a	119.2±1.2a	121.6±0.9a	106.0±1.0b
	4	112.3±4.2ab	112.0±1.8ab	116.4±1.9ab	120.1±0.9a	108.0±1.4b
	5	116.2±2.4	109.0±3.6	114.1±5.1	121.2±3.0	103.0±6.0
	6	98.3±2.7	104.2±5.6	106.0±2.5	113.7±5.0	101.0±1.1

	1	33.5±0.8c	38.5±0.9b	39.6±0.4ab	41.4±0.2a	37.8±0.0b
	2	41.5±2.0a	42.3±0.8a	42.6±0.7a	42.2±0.5a	36.1±0.6b
VSL (μm/s)	3	36.1±1.3bc	39.0±0.9ab	39.5±0.6ab	41.0±0.3a	34.6±0.4c
	4	35.5±1.6ab	36.4±1.3ab	36.8±0.8ab	39.8±0.2a	34.6±0.9b
	5	37.2±1.9	34.4±2.1	34.9±2.2	37.4±1.0	31.0±1.8
	6	27.6±0.4	32.5±2.0	31.5±1.7	34.5±2.0	28.5±0.8
	1	64.0±1.8b	63.2±0.7b	63.4±0.4b	75.5±0.6a	63.5±3.0b
	2	65.5±3.1ab	67.4±0.6a	68.9±0.0a	69.1±1.3a	57.8±1.7b
VAP (μm/s)	3	56.0±1.1bc	61.2±1.2a	60.2±1.1ab	62.5±0.5a	52.3±0.9c
	4	55.0±1.8bc	55.6±0.9bc	57.3±1.1ab	61.1±0.6a	51.5±0.8c
	5	56.9±1.5ab	53.1±2.3ab	55.1±2.4ab	59.9±1.7a	48.5±3.0b
	6	45.1±1.4b	50.1±2.9ab	52.0±1.2ab	56.2±2.2a	46.4±0.6b
	1	54.6±0.2ab	60.2±0.5a	60.0±0.5a	53.8±0.6b	60.1±2.8a
	2	62.2±0.6	60.8±0.6	60.0±0.7	61.0±1.0	62.9±1.4
STR (%)	3	61.9±2.1	60.3±0.5	63.9±0.5	64.2±0.3	64.3±0.5
	4	61.7±1.8	62.7±1.3	61.9±0.7	64.2±0.8	64.2±0.6
	5	61.9±1.7	62.3±1.3	61.6±1.9	60.7±0.0	61.5±1.6
	6	58.6±2.0	62.4±0.7	58.8±1.8	59.1±1.4	57.6±0.5
	1	28.8±0.3ab	31.1±0.1a	31.0±0.3a	27.3±0.3b	31.3±1.6a
	2	32.8±0.5a	31.1±0.2ab	30.3±0.2b	31.2±0.4ab	32.6±0.7a
LIN (%)	3	31.9±0.9	31.0±0.3	32.6±0.5	33.3±0.2	32.2±0.3
	4	30.6±1.4	31.5±0.9	30.8±0.5	33.0±0.4	30.9±0.4
	5	30.7±1.2	30.7±0.9	30.1±0.7	30.2±0.1	28.9±0.5
	6	27.2±0.7bc	30.4±0.4a	29.1±0.8abc	29.7±0.5ab	27.1±0.3c
WOB (%)	1	51.3±0.1a	50.4±0.4ab	49.8±0.4ab	49.5±0.3b	50.9±0.6ab
	2	51.3±0.5a	49.6±0.2bc	49.1±0.4c	50.1±0.3abc	50.7±0.1ab

	3	50.3±0.2ab	49.9±0.2ab	49.8±0.7ab	50.8±0.1a	48.8±0.6b
	4	48.3±0.7bc	49.0±0.3ab	48.5±0.3bc	50.5±0.1a	46.8±0.3c
	5	48.0±0.3a	48.0±0.5a	47.8±0.2a	48.7±0.2a	45.9±0.3b
	6	45.3±0.4c	47.3±0.2b	48.6±0.2ab	48.9±0.4a	45.6±0.1c
ALH (μm)	1	7.7±0.1c	8.6±0.1ab	8.5±0.1abc	9.1±0.1a	8.0±0.3bc
	2	7.9±0.2ab	8.7±0.1a	8.6±0.1ab	8.7±0.3a	7.5±0.4b
	3	7.8±0.0cd	8.6±0.1a	8.1±0.2bc	8.4±0.1ab	7.4±0.0d
	4	7.7±0.3ab	7.8±0.2ab	8.2±0.1a	8.2±0.1a	7.4±0.1b
	5	7.6±0.1bc	7.8±0.1bc	8.0±0.1ab	8.7±0.1a	7.2±0.3c
	6	7.1±0.3c	7.6±0.3abc	8.3±0.1ab	8.4±0.1a	7.4±0.1bc
BCF (Hz)	1	36.2±0.3	35.9±0.1	36.0±0.1	34.8±0.4	35.2±0.6
	2	35.2±0.5	35.2±0.3	35.3±0.4	33.9±1.2	35.4±0.4
	3	33.6±1.4	34.0±0.5	33.0±1.3	34.7±0.5	35.2±1.0
	4	35.3±1.0ab	34.1±0.1bc	33.0±0.8bc	32.3±0.2c	37.0±0.4a
	5	37.5±0.4	36.5±1.9	35.6±2.4	35.7±0.7	39.3±2.2
	6	38.5±1.5ab	39.8±0.8a	32.3±2.1b	34.0±1.5ab	36.1±0.9ab

Data are presented as mean ± SEM from five independent pooled semen collections ($n = 3$). CASA fields at each incubation time were averaged within each biological replicate before statistical analysis. Values within the same row bearing different superscript lowercase letters differ significantly among treatments at the same incubation time ($p < 0.05$).
